# Dual Transgene Expression in Murine Cerebellar Purkinje Neurons by Viral Transduction *In Vivo*

**DOI:** 10.1371/journal.pone.0104062

**Published:** 2014-08-05

**Authors:** Marie K. Bosch, Jeanne M. Nerbonne, David M. Ornitz

**Affiliations:** 1 Developmental Biology, Washington University School of Medicine, Saint Louis, Missouri, United States of America; 2 Internal Medicine, Washington University School of Medicine, Saint Louis, Missouri, United States of America; University of Iowa Carver College of Medicine, United States of America

## Abstract

Viral-vector mediated gene transfer to cerebellar Purkinje neurons *in vivo* is a promising avenue for gene therapy of cerebellar ataxias and for genetic manipulation in functional studies of animal models of cerebellar disease. Here, we report the results of experiments designed to identify efficient methods for viral transduction of adult murine Purkinje neurons *in vivo.* For these analyses, several lentiviral and an adeno-associated virus (AAV), serotype 1, vector with various promoter combinations were generated and compared for *in situ* transduction efficiency, assayed by fluorescent reporter protein expression in Purkinje neurons. Additional experiments were also conducted to identify the optimal experimental strategy for co-expression of two proteins in individual Purkinje neurons. Of the viruses tested, AAV1 with a CAG promoter exhibited the highest specificity for Purkinje neurons. To deliver two proteins to the same Purkinje neuron, several methods were tested, including: an internal ribosome entry site (IRES), a 2A sequence, a dual promoter vector, and co-injection of two viruses. Efficient expression of both proteins in the same Purkinje neuron was only achieved by co-injecting two AAV1-CAG viruses. We found that use of an AAV1-CAG virus outperformed similar lentivirus vectors and that co-injection of two AAV1-CAG viruses could be used to efficiently deliver two proteins to the same Purkinje neuron in adult mice. AAV1 with a CAG promoter is highly efficient and selective at transducing adult cerebellar Purkinje neurons and two AAV-CAG viruses can be used to efficiently express two proteins in the same neuron *in vivo*.

## Introduction

The cerebellum functions as an important regulator of body movement and coordination, and, as such, disorders of the cerebellum typically result in ataxia, a clinical symptom characterized by lack of coordination and gait disturbances. As the sole output of the cerebellar cortex, Purkinje neurons are critical for cerebellar information processing [1] and, therefore, may provide an ideal target for therapies designed to restore or improve cerebellar function. Cerebellar disease can be acquired (e.g. ethanol, drugs, stroke, or trauma) or inherited [2]. The inherited cerebellar ataxias, many of which have known genetic bases, may be particularly amenable to treatments based on gene transfer. Studies of cerebellar function and pathophysiology would also benefit from methods to genetically manipulate Purkinje neurons in adult model organisms. Viral vectors provide a promising gene delivery system in both basic research and gene therapy.

For some gene transfer applications, it is desirable to express more than one protein in a given cell. Such situations may include transfer of multiple genes that cooperate functionally or transfer of a gene of interest with a fluorescent reporter gene for easy identification of transduced cells in living systems. A number of strategies have been used successfully to co-express multiple transgenes in the same cell using viral vectors, including internal ribosome entry sites (IRES) elements [3], 2A peptides [4], dual internal promoters [5], and co-infection with multiple viral vectors [6]. One of the most commonly used approaches has been insertion of an IRES element between two transgenes, which enables production of two polypeptides from a single transcript [7]. More recently, however, 2A peptides have gained in popularity due to their small size (∼18–22 amino acids) and ability to produce discrete proteins in essentially equimolar quantities [8]. Insertion of a 2A sequence between two genes results in ribosomal ‘skipping’ during translation [9], such that the ribosome continues downstream without formation of a peptide bond.

The goal of this study was to develop a viral vector system for efficient and selective virally transduced gene expression in adult murine Purkinje neurons *in vivo* and to optimize for co-expression of two transgenes. As an example, we used Fibroblast Growth Factor 14 (FGF14), which is highly expressed in wild type cerebellar Purkinje neurons [10], [11] and in which a mutation causes Spinocerebellar ataxia, type 27 (SCA27) in humans [12]–[15]. We tested four lentiviral and one AAV construct with various promoter and fluorescent reporter gene combinations and found optimal expression in adult murine Purkinje neurons in vivo using AAV serotype 1 (AAV1) constructs containing a modified chicken β-actin promoter with the CMV-IE enhancer (CAG) [16]. Co-expression of FGF14 with a fluorescent reporter gene in the same neuron in adult mice was only achieved by co-injecting two AAV1 viruses both of which contained CAG promoters to drive expression.

## Materials and Methods

### Ethics Statement

This study was performed in accordance with guidelines from the NIH Guide for the Care and Use of Laboratory Animals, and all protocols involving animals were approved by the Washington University Animal Studies Committee. All surgery was performed under isofluorane gas or ketamine/xylazine anesthesia, and every effort was made to minimize pain and suffering.

### Mice

Adult (2–4 months) wild type C57BL/6, *Fgf14^−/−^* [17], and L7/pcp2-GFP [18] mice were maintained in accordance with guidelines from the NIH Guide for the Care and Use of Laboratory Animals, and all protocols involving animals were approved by the Washington University Animal Studies Committee. Genotypes were confirmed by PCR analysis.

### Plasmid constructs

Lentiviral vectors used in this study were constructed from third-generation self-inactivating (SIN) lentiviral transfer vectors. The plasmid constructs for lentiviral vectors MND-GFP, PGK-GFP, and UBC-Venus have been described previously [19]. MSCV-GFP (SIN-MU3-EGFP-W), in which eGFP expression is controlled by a murine stem cell virus (MSCV) LTR [20], was provided by R. Hawley (George Washington University). AAV-IRES-GFP (pTR-UF-12.1) has been described previously [21].

Lentivirus construct for MND-GFP (pCCL-cppt-MNDU3-GFP) expresses eGFP under control of a modified Moloney murine leukemia virus (MoMuLV) LTR with myeloproliferative sarcoma virus enhancer, deleted negative control region, and substituted Δl587rev primer-binding site [22], [23]. PGK-GFP (pRRLsinPGKGFPppt), contains a human phosphoglycerate kinase (PGK) promoter followed by eGFP [24]. In UBC-Venus (FCIV.FM1), Venus (a yellow fluorescent protein) expression is controlled by a ubiquitin C promoter and internal ribosome entry site (IRES).

**Figure 1:**
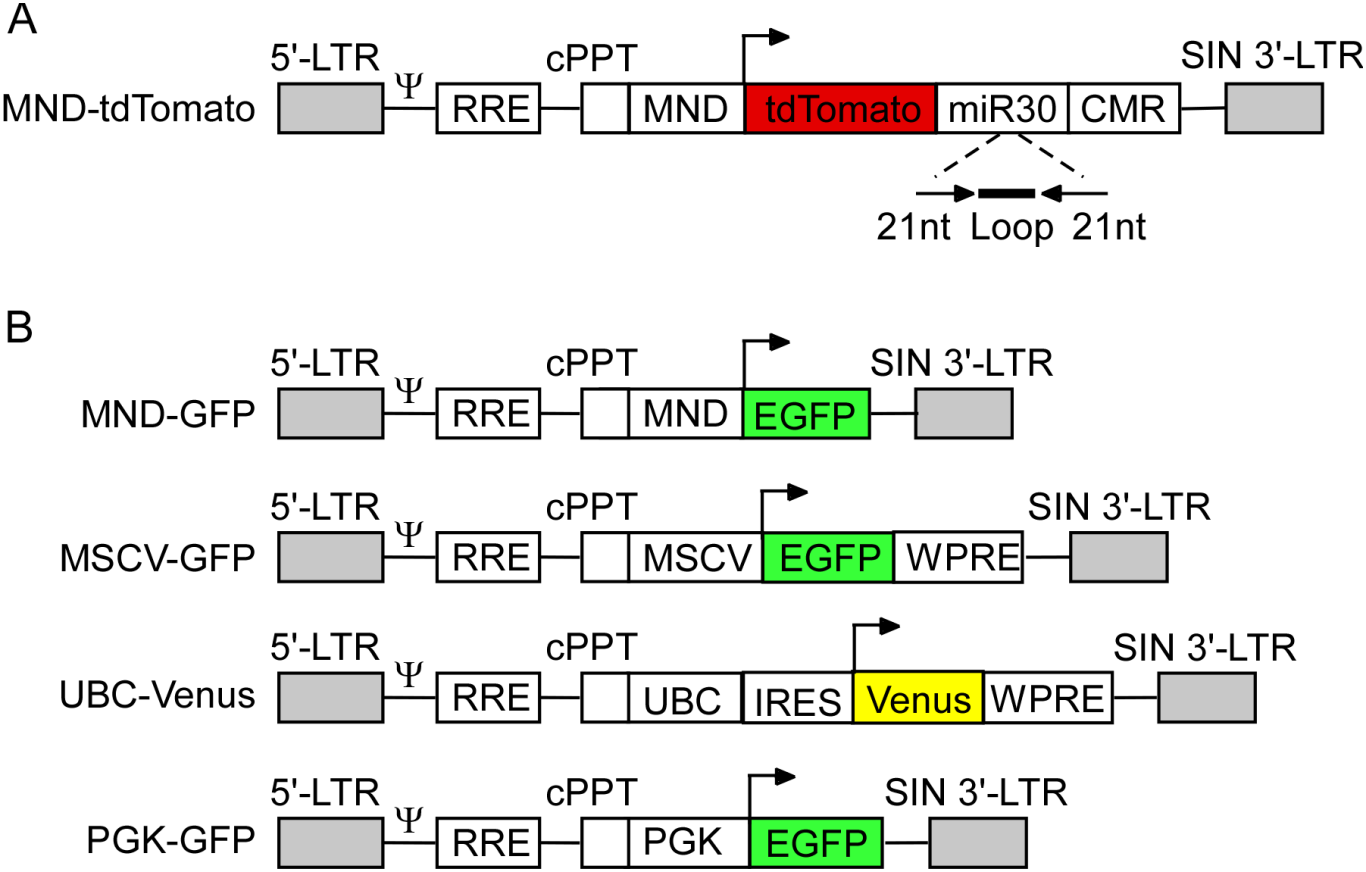
Schematic representation of lentiviral constructs. A–B. Schematic of the lentiviral constructs used in this study. Arrows represent mammalian transcriptional start sites. In all vectors, the 3′-LTR contains a deletion in the U3 region which renders the virus self-inactivating (SIN 3′-UTR) and replication incompetent. A. The MND-tdTomato lentiviral vector contains a microRNA (miR30) and chloramphenicol resistance gene (CMR) in the 3′-UTR of the tdTomato fluorescent reporter gene. B. The internal promoters are MND, modified MoMuLV LTR containing myeloproliferative sarcoma virus enhancer; MSCV, murine stem cell virus LTR; UBC, Ubiquitin C promoter; and PGK, human phosphoglyercerate kinase promoter. Ψ: packaging signal; RRE, REV response element; cPPT, central polypurine tract; EGFP, enhanced green fluorescent protein; IRES, internal ribosome entry site; WPRE, woodchuck hepatitis virus posttranscriptional regulatory element; LTR, long terminal repeat.

The lentiviral vector MND-tdTomato (pMRT-tdTomato-shRNAmir) was constructed by linearizing the lentiviral MND vector (pCCL-cppt-MNDU3) with EcoRI and SalI and inserting a new polylinker containing NheI and PacI restriction enzyme sites. The polylinker was synthesized as single-stranded DNA oligonucleotides by IDT (Coralville, IA). Oligonucleotides were annealed together prior to insertion into MND vector. The DNA sequence of the new polylinker in the MND vector (pMND-NP) was 5′-GAATTGGCTAGCGTTAACGGATCCGCTTAATTAAGTACGCGTCCCGGGGTCGAC-3′, which corresponds to the restriction enzyme sequence of ClaI-NheI-HpaI-BamHI-PacI-MluI-SmaI-SalI-BclI-KpnI, which maintained the SalI restriction site but destroyed the EcoRI site. pMND-NP was then linearized by digesting with NheI and PacI and a tdTomato-shRNAmir-CMR (chloramphenicol resistance gene) cassette was inserted after being cut out with the same enzymes. The insert was prepared from pGEM-pPRIME-CMV, which was made by moving the CMV-GFP-shRNAmir-CMR cassette from pPRIME-CMV-GFP vector into pGEM vector and replacing the GFP fluorophore with tdTomato. pPRIME-CMV-GFP was a gift from S. Elledge (Harvard University) and contains an shRNAmir in the 3′-UTR of the GFP fluorescent reporter gene. The shRNAmir is an shRNA sense-loop-antisense sequence with 19 nt loop embedded in a mir30 microRNA context [25] (Figure 1A). The final MND-tdTomato (pMRT-tdTomato-shRNAmir) vector consisted of the MND promoter followed by tdTomato fluorescent reporter gene, shRNAmir, and CMR. The 21-mer shRNA sequence is 5′-GCCATTGGAAGTTGCCATGTA-3′, and targets murine *Fgf14*. The insert was verified by sequencing before packaging into virus.

AAV-IRES-GFP (pTR-UF-12.1) [21] consists of a CMV early enhancer with chicken β-actin (CAG) promoter followed by a simian virus 40 (SV40) intron, internal ribosome entry site (IRES), eGFP, SV40 polyadenylation (polyA) site and bovine growth hormone (BGH) polyA site. To generate, AAV-GFP without IRES, AAV-CAG-hFGF14B-P2A-GFP (see below) was digested with NsiI and MluI to remove the hFGF14B-P2A-GFP cassette. GFP was amplified from pQBI-fC2 (Quantum Biotechnology Inc., Montreal, Canada) to add a 5′-NsiI restriction site and Kozak consensus sequence upstream of the initiation methionine and a downstream 3′-Mlu and translational stop codon. PCR fragment was digested with NsiI and MluI and inserted into the digested AAV vector. The resulting construct contained the CAG promoter followed by the SV40 intron, GFP, SV40 polyA, and BGH polyA. The plasmid was verified by sequencing and was transfected into CHL1610 cells to confirm expression of the GFP reporter gene before viral particle packaging.

To construct AAV-FGF14B-GFP (fusion), AAV-IRES-GFP was digested with NotI to remove GFP, and an MluI linker with NotI sticky ends was inserted in its place (IDT, Coralville, IA). The AAV plasmid without GFP was then cut with EcoRV (blunt) and MluI to remove the IRES. The hFGF14B-GFP insert was prepared by digestion of pQBI-hFGF14B-GFP plasmid [26] with ApaI followed by blunting with Klenow. Blunted plasmid DNA was then cut with MluI, and the hFGF14B-GFP fragment was gel purified. The hFGF14B-GFP insert with a 5′-blunt end and 3′-MluI sticky end was then ligated into the AAV plasmid with blunt and MluI sticky end sites. The final AAV-hFGF14B-GFP construct consisted of the CAG promoter followed by the SV40 intron, hFGF14B-GFP fusion, SV40 polyA, and BGH polyA. Before viral packaging, the insert was verified by sequencing, and fluorescent reporter expression was verified by transfection into CHL1610 cells.

To create AAV-FGF14B-IRES-tdTomato, AAV-IRES-tdTomato was used. AAV-IRES-tdTomato was created from AAV-IRES-GFP (pTR-UF-12.1) by replacing the GFP with tdTomato. AAV-IRES-tdTomato contains the CAG promoter followed by the SV40 intron, IRES, tdTomato, SV40 polyA, and BGH polyA. hFGF14B was amplified by PCR from pQBI-hFGF14B-GFP to add a 5′-SpeI restriction site and 3′ translational stop codon and NsiI restriction site. PCR fragment was digested with SpeI and NsiI and inserted into AAV-IRES-tdTomato also digested with SpeI and NsiI. The resulting AAV-hFGF14B-IRES-tdTomato construct contained the CAG promoter followed by the SV40 intron, hFGF14B, IRES, tdTomato, SV40 polyA, and BGH-polyA. The insert was verified by sequencing, and plasmid DNA was transfected into CHL1610 cells to confirm expression of the tdTomato fluorescent reporter prior to viral packaging.

To generate P2A constructs, oligonucleotides containing the P2A sequence with 5′-NotI and 3′-NheI sticky ends were synthesized (IDT, Coralville, IA). A Gly-Ser-Gly linker amino acid sequence was also added at the 5′ end. The following GSG-P2A oligonucleotide sequence was used: 5′-GGAAGCGGAGCTACTAACTTCAGCCTGCTGAAGCAGGCTGGAGACGTGGAGGAGAACCCTGGACCT-3′ [27], which corresponds to the peptide sequence: GSG-ATNFSLLKQAGDVEENPG•P, with • representing the point of cleavage. AAV-hFGF14B-GFP was cut with NotI and NheI to open the vector between the hFGF14B and GFP sequences, and the annealed P2A oligonucleotide was inserted. AAV-hFGF14B-P2A-GFP plasmid contained the CAG promoter followed by the SV40 intron, hFGF14B, P2A, GFP, SV40 polyA, and BGH polyA. The P2A insert was verified by sequencing. Before viral particle generation, GFP fluorescence was confirmed by transfection of plasmid DNA into CHL1610 cells, and GFP cleavage was validated by Western blotting.

To generate dual promoter AAV plasmids, the IRES-GFP cassette was first removed from the AAV-IRES-GFP (pTR-UF-12.1) plasmid by cutting with NotI to remove the GFP and blunting the ends with Klenow. AAV-IRES linearized plasmid was then cut with EcoRV to remove the IRES and create a blunt site following the CAG promoter. Blunt ends were then ligated together to generate an AAV-CAG-empty plasmid consisting of the CAG promoter followed by the SV40 intron, SV40 polyA, and BGH-polyA. The AAV-CAG-empty vector was then digested with SalI to linearize the plasmid between the SV40 polyA and BGH-polyA sequences. Digested SalI sticky ends were dephosphorylated with calf intestinal phosphatase (CIP) followed by column purification (QIAquick PCR purification kit, Qiagen). The PGK-GFP insert was prepared from pRRLsinPGKGFPppt by digesting with XhoI and SalI and was directly ligated into The AAV-CAG-empty vector digested with SalI to create AAV-CAG-empty-PGK-GFP. Correct orientation of PGK-GFP insert was verified by restriction digest. AAV-CAG-empty-PGK-GFP was then cut with NsiI and ClaI to open the vector between the SV40 intron and SV40 polyA. mFGF14A insert was prepared by PCR from pQBI-mFGF14A-GFP [26] to add a 5′-NsiI restriction site and Kozak consensus sequence upstream of the initiation methionine and 3′-ClaI restriction site and a translational stop codon. The mFGF14A PCR product was inserted into pCR2.1-TOPO-TA vector (Invitrogen), and then digested with NsiI and ClaI. The mFGF14A fragment was then inserted into AAV-CAG-empty-PGK-GFP vector to make the AAV-CAG-mFGF14A-PGK-GFP dual promoter vector. The final plasmid construct thus contained the CAG promoter followed by the SV40 intron, mFGF14A and SV40 polyA and the PGK promoter followed by GFP and BGH polyA. Inserts were verified by sequencing, and GFP expression was verified by transfection into CHL1610 cells.

### Lentiviral vector production

Lentiviral viral vectors were generated by the Hope Center Viral Vectors Core as described previously [28]. Briefly, the packaging cell line, HEK293T was maintained in Dulbecco’s modified Eagles medium (DMEM), supplemented with 10% fetal bovine serum (FBS), 100 units/ml penicillin, 100 µg/ml streptomycin in a 37°C incubator with 5% CO_2_. HEK293T cells were plated at 30–40% confluence 24 h before transfection (70–80% confluence when transfected). Ten µg of lentiviral vector with the appropriate insert, 5.8 µg of pMD-Lg, 3.1 µg of pCMV-G, and 2.5 µg of RSV-REV were co-transfected into 293T cells using the calcium phosphate precipitation procedure. Six hours after transfection, the medium was replaced with the complete medium containing 6 mM sodium butyrate. Culture supernatant was collected 42 h after transfection. The supernatant was passed through a 0.45 µm filter, concentrated by ultracentrifugation through a 20% sucrose cushion, and stored at −80°C until use. Vector titers were determined by transduction of HT1080 cells and assayed for reporter expression using flow cytometry. The lentiviral titers used in this study are as follows: PGK-GFP = 1×10^8^ TU/ml; UBC-Venus = 5.4×10^7^ TU/ml; MND-GFP = 4.7×10^9^ TU/ml; MSCV-GFP = 1.5×10^8^ TU/ml; MND-tdTomato-sh4 (referred to as MND-tdTomato) = 1.8×10^9^ TU/ml.

### AAV vector production

AAV viral vectors were generated by the Hope Center Viral Vectors Core as described previously [29]. Briefly, HEK293 cells, maintained as above were plated at 30–40% confluence in CellSTACS (Corning, Lowell, MA) 24 h before transfection (70–80% confluence when transfection). 1.8 mg helper plasmid (e.g. pXYZ1 for AAV1) and 0.6 mg rAAV transfer plasmid containing the gene of interest were co-transfected using the calcium phosphate precipitation procedure. Cells were incubated at 37°C for 3 days before harvesting. Cells were lysed by three freeze/thaw cycles. The cell lysate was treated with 50 U/ml of Benzonaze followed by iodixanol gradient centrifugation. The iodixanol gradient fraction was further purified by column chromatography using HiTrap Q columns (GE Healthcare) for AAV1. The eluate was concentrated with Vivaspin 20 100K concentrator (Sartorius Stedim, Bohemia, NY). Viral titer was determined by dot blot assay. In the dot blot assay, AAV viral prep was treated with DNaseI to remove DNA that was not in the viral particle. After inactivating the DNaseI, vector genome was released from viral particles by digestion with proteinase K. DNA was extracted, denatured, and transferred to nylon membranes. A serially diluted AAV plasmid with known copy number was also transferred to membrane. A ^32^P-labeled oligonucleotide probe containing the sequence in the AAV vector was hybridized to membranes and signal was detected by exposure to X-ray film. Titer was calculated by comparison with standard curve of AAV plasmid with known copy number. Titers of AAV viruses used in this study were as follows: AAV1-CAG-GFP 7.1×10^12^ vg/ml (viral genomes per ml); AAV1-CAG-FGF14B-GFP = 1.7×10^13^ vg/ml; AAV1-CAG-FGF14B-IRES-tdtomato = 5×10^12^ vg/ml; AAV1-CAG-FGF14B-P2A-GFP = 5×10^12^ vg/ml; and AAV1-dual promoter-CAG-mFGF14A-PGK-GFP = 1×10^13^ vg/ml.

### Cell culture, transfection, and western blots

Chinese hamster lung (CHL) 1610 cells [30] were maintained in RPMI media supplemented with 10% fetal bovine serum (Gibco) and 100 U/ml penicillin, 100 µg/ml streptomycin, and incubated at 37°C with 5% CO_2_. Cells were transfected at 80–90% confluency using Lipofectamine 2000 (Invitrogen) according to the manufacturer’s instructions. 5 µg of plasmid DNA was transfected into cells plated in 60 mm^2^ dishes. 24 h after transfection, fluorescence was visualized and photographed in living cells using a Leica DMIL LED inverted microscope. Following visualization, cells were washed once with 2 ml ice cold PBS and lysed in ice cold lysis buffer (20 mM Tris-HCl, 150 mM NaCl, and 1% NP-40). Protease inhibitor mixture (Protease Inhibitor Cocktail Set III; Millipore, Bedford, MA) was added immediately before cell lysis. Cell extracts were collected, incubated with slow rotation for 15 min at 4°C, and centrifuged for 10 min at 3000 rpm and 4°C to remove the insoluble fraction. Protein content was measured using a BCA assay kit (Pierce-Thermo, Rockford, IL). For Western blots, 50 mM Bond-Breaker TCEP (Thermo Fisher Scientific, Rockford, IL) was added to cell lysates and 30 µg of each lysates were loaded into each lane. Resolved proteins were transferred to PVDF-P membranes (Millipore, Bedford, MA) for 1.5 h at 4°C and blocked with 3% non-fat dry milk (vol/vol) in PBST. Blots were probed with primary antibody diluted in 0.5% non-fat dry milk (vol/vol) in PBST for 2 h at room temperature. After washing extensively with PBST, blots were incubated with secondary antibody diluted in 0.5% non-fat dry milk (vol/vol) in PBST for 1 h at room temperature. The primary antibodies used for western blotting were mouse monoclonal anti-GFP (1∶1000, NeuroMab, N86/8) and rabbit polyclonal anti-FGF14 (1∶1000). Secondary antibodies used were goat anti-mouse-HRP or goat anti-rabbit-HRP (both 1∶5000, Santa Cruz Biotechnology, Dallas, TX).

### Stereotactic injections

Mice were anesthetized with isoflurane gas (2%) or ketamine/xylazine cocktail (30 mg/ml ketamine and 4 mg/ml xylazine, at a dose of 1 ml/kg, i.p.) and fixed in a stereotactic frame (David Kopf, Tujunga, California or Stoelting, Wood Dale, Illinois). For lentiviral injections, a midline incision was made on the scalp, and a 2 mm burr hole was made using a dental drill, and a 5 ml Hamilton syringe was lowered 0.25–0.5 mm below the dural membrane at the previously determined coordinates. A nanoinjector pump (Stoelting) was used for infusion of 3–6 µl virus at a rate of 0.1–0.2 µl/min, after which the needle was left in place for 5–10 min to ensure complete diffusion of the virus. At the end of the injection, the incision was closed with a 4–0 nylon suture and triple antibiotic ointment was applied topically. For some lentiviral and all AAV injections, the cranium at the desired coordinates was thinned, and a craniotomy was performed by using a scalpel to gently lift a small flap of bone away from the surface of the brain. Virus was loaded into pulled glass pipettes (outer tip diameter of 18–25 µm) and injected using a Picospritzer (Parker Hannifin, Mayfield Heights, Ohio). Approximately 1–2 µl of virus was injected over a 5–10 min period. Following virus injection, incision was closed using surgical staples. Mice were placed into a warming chamber until consciousness was fully regained. The sterotaxic coordinates to target the Purkinje neuron layer of lobule VI were: midline, 1–2 mm anterior to interparietal-occipital suture (or about 5–6 mm caudal to bregma) and 0.35 mm below the pial surface. For each virus, two or more animals were injected per experiment, and representative images were chosen. The exact n for each virus condition is given in Table 1. We did not see a correlation between the viral titer and the ability of the viral prep to label any cell near the injection site.

**Table 1:** Purkinje neuron transduction by lentiviruses and AAV.

Virus	Animalsinjected	Purkinjeneurontransduction^a^	Other celltypes transduced^b^
Lv-PGK-GFP	2	+	WM
Lv-UBC-Venus	2	-	GCL
Lv-MSCV-GFP	2	-	BG
Lv-MND-GFP	2	-	BG, WM
Lv-MND-tdTomato	6	-	BG, WM
AAV1-CAG-GFP	13	++	ML
AAV1-CAG-FGF14B-GFP	6	++	ML
AAV1-CAG-FGF14B-IRES-tdTomato	16	++	ML
AAV1-CAG-FGF14B-P2A-GFP	2	++	ML
AAV1-CAG-FGF14A-PGK-GFP	4	++	CAG-FGF14 in ML,PGK-GFP in BG

a -,≤1 Purkinje neurons transduced per 20x field; +, ≤5 Purkinje neurons transduced per 20x field; ++, >5 Purkinje neurons transduced per 20x field.

b Cells transduced based on cell morphology and cerebellar region. GCL, granule cell layer; BG, Bergmann glia; WM, white matter; ML, molecular layer.

### Immunostaining

7–14 days following lentivirus injection or 4–8 weeks following AAV virus injection, mice were deeply anesthetized with a ketamine/xylazine cocktail (30 mg/ml ketamine and 4 mg/ml xylazine, at a dose of 1 ml/kg, i.p.) and transcardially perfused with 0.9% NaCl and followed by ice-cold fixative, consisting of 1% formaldehyde freshly prepared from paraformaldehyde powder [31] in 0.1 M phosphate buffer, pH 7.4. Brains were removed and post-fixed for one hour in the same fixative at 4°C, followed by overnight cryoprotection in 30% sucrose in 0.1 M phosphate buffer at 4°C. Brains were embedded in OCT, and sagittal cryostat sections of cerebellum (16 µm) were mounted onto slides and stored at −80°C until processing.

All of the following steps were at room temperature. For examining fluorescent reporter gene expression without immunostaining, slides containing cerebellar sections were rinsed twice in 0.01 M phosphate buffered saline (PBS), pH 7.4 (Sigma, St. Louis, MO) and coverslips were mounted with Vectashield mounting medium (Vector Laboratories). For immunostaining, slides were washed twice with PBS and permeabilized for 20 min in PBS with 0.1% Triton X-100 (vol/vol). Sections were incubated with blocking solution (PBS plus 10% goat serum) for 1 h, followed by staining overnight with primary antibodies diluted in PBS with 0.1% Triton X-100 and 0.1% bovine serum albumin. After washing with PBS, sections were incubated with appropriate goat secondary antibodies conjugated to Alexa 488 or 594 or 657 (1∶400, Invitrogen) diluted in PBS for 1 h. Sections were again washed with PBS and coverslips were mounted using Vectashield Hardset mounting medium (Vector Laboratories) and allowed to dry overnight at 4°C. The following primary antibodies were used: mouse monoclonal anti-FGF14 (1∶1000, NeuroMab, clone N56/21), mouse monoclonal anti-AnkyrinG (1∶1000, NeuroMab, clone N106/36), and mouse monoclonal anti-GFP (1∶1000, NeuroMab, clone N86/38). The FGF14 antibody has been validated using *Fgf14^−/−^* mice [10], [11]. The AnkyrinG antibody is a validated NeuroMab antibody and in our experience it gives very specific staining of the AIS. The GFP antibody is a validated NeuroMab antibody and does not give background staining with wild type mouse tissue. The Images were captured using a confocal laser scanning microscope (Olympus Fluoview-500) using a 20x or 60x oil-immersion objective. Sequential acquisition of multiple channels was used, and z-stacks were collected at 0.5 µm steps. Image stacks were converted into maximum intensity z-projections using ImageJ software (NIH).

## Results

### Patterns of cellular transduction by lentiviral vector with MND promoter

**Figure 2:**
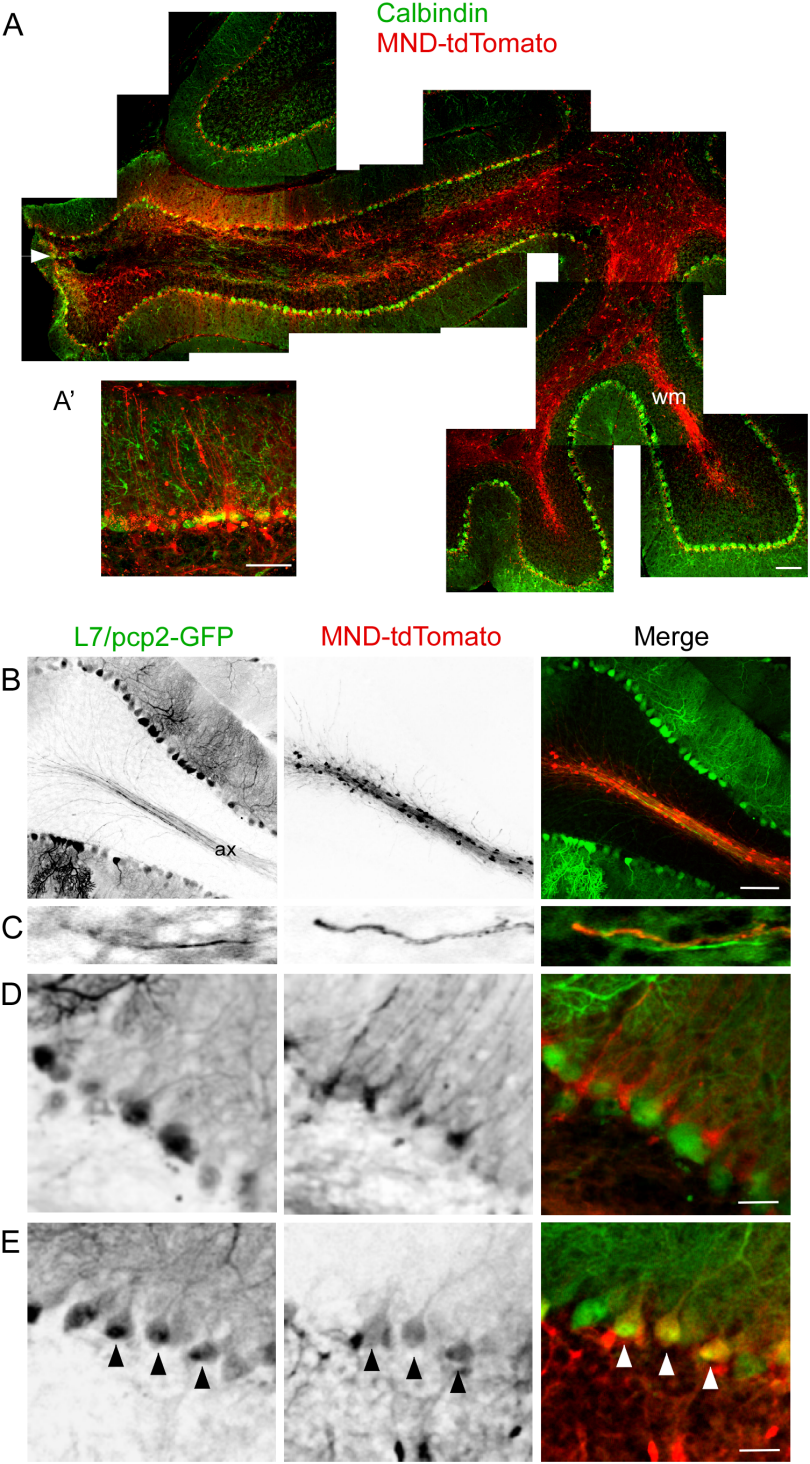
Patterns of cellular transduction by Lenti-MND-tdTomato. A. Montage of low magnification confocal images of sagittal sections of wild type mouse cerebellum injected with MND-tdTomato (red) and immunostained for calbindin (green), a marker for Purkinje neurons. A region of tdTomato expressing cells in the Purkinje and/or molecular layer is visible near the injection site, but the vast majority of tdTomato-expressing cells are in the white matter (wm). Arrowhead indicates approximate location of injection, where some damage to brain parenchyma can be seen. A’. A higher magnification image from an adjacent slide illustrating the lack of co-localization of tdTomato-expressing processes and calbindin positive Purkinje neuron dendrites. B–E. To examine transduction patterns in more detail, MND-tdTomato was injected into L7/pcp2-GFP mouse cerebellum, and sagittal sections were examined at higher magnification. L7/pcp2-GFP mice express GFP under control of the Purkinje cell specific promoter L7/pcp2. B, GFP expression is visible in Purkinje neuron somata, dendrites, and axons (ax), which project into the white matter tract (left panel and green, right panel). tdTomato-expressing somata are located in the white matter tracts and extend short processes (center panel and red, right panel). C. Purkinje neuron axons (left panel and green, right panel) and processes expressing tdTomato (center panel and red, right panel) do not overlap. D-E. High magnification images of the Purkinje layer of L7/pcp2-GFP cerebellum injected with MND-tdTomato. D. GFP-expressing Purkinje neuron somata with characteristic highly branched dendrites (left and green, right panel). Cells expressing tdTomato are located in the Purkinje layer but somata are smaller and processes are straight and unbranched (center). tdTomato (red) expression pattern does not colocalize with GFP (green) expressing Purkinje neuron somata or dendrites (right panel). The shape and location of tdTomato expressing cells is consistent with Bergmann glia. E. Coexpression of GFP (left) and tdTomato (center) in three Purkinje neuron somata (arrowheads, and yellow, right panel). Scale bars: 100 µm (A, B), 50 µm (A’), 25 µm (D, E).

To explore methods to efficiently transduce Purkinje neurons *in vivo*, third generation self-inactivating (SIN) vectors with various combinations of promoters and fluorescent reporters genes were constructed as diagrammed in Figure 1A–B. Initial experiments to target Purkinje neurons *in vivo* used a lentiviral construct expressing tdTomato under control of the MND promoter (modified Moloney murine leukemia virus (MoMuLV) LTR with myeloproliferative sarcoma virus enhancer) (Figure 1A). This construct also contains an shRNA in the 3′-UTR of the tdTomato gene, but only viral transduction was evaluated in these experiments. MND-tdTomato viral particles were injected directly into wild type mouse cerebellum, and sections were immunostained with an anti-calbindin antibody to label Purkinje neurons. Consistent with previous reports [32], calbindin immunoreactivity was evident in Purkinje neuron somata (Figure 2A). Examination of sagittal cerebellar sections at low magnification revealed a moderate amount of damage to the brain parenchyma near the injection site (Figure 2A, arrowhead). Bright tdTomato expression was localized mainly to cerebellar white matter and spread far from the injection site into adjacent lobes (Figure 2A, Table 1). The Purkinje and/or molecular layers exhibited tdTomato expression only in a small region. A higher magnification image of this region in an adjacent slide (Figure 2A’) illustrates a lack of co-localization of tdTomato-expressing and calbindin-expressing processes.

To investigate the cell types transduced with MND-tdTomato in more detail, lentiviral particles were injected into L7/pcp2-GFP mice, which express GFP under control of the Purkinje cell specific promoter, L7/pcp2 [18]. Purkinje neuron cell bodies, dendrites and axons are clearly labeled with GFP in L7/pcp2 mice (Figure 2B–E). In cerebellar white matter tracts, GFP expression was visible in Purkinje neuron axons, whereas tdTomato expression was evident in discrete punctae and short processes radiating towards the Purkinje layer (Figure 2B). Interestingly, tdTomato expression in these short processes did not co-localize with GFP expression in Purkinje neuron axons (Figure 2C). Together, this suggested that MND-tdTomato transduced cells in the white matter were myelinating glia and not Purkinje neuron axons (Table 1). Closer examination of the Purkinje layer revealed that most of the transduced cells in that region were Bergmann glia, with small cell bodies in the Purkinje layer and radial processes extending into the molecular layer (Figure 2D, Table 1). In rare instances, transduced Purkinje neurons were observed (Figure 2E, arrowheads).

### Comparison of lentiviral vectors with various promoters

To compare promoter activity in adult Purkinje neurons in vivo, we tested lentiviral constructs expressing EGFP or Venus fluorescent reporter genes under control of various promoters (Figure 1B). In addition to the MND promoter described in Figure 2, the following promoters were tested: MSCV (LTR from murine stem cell virus); Ubiquitin C (UBC); and PGK (human phosphoglycerate kinase promoter). Lentiviruses were injected directly into wild type mouse cerebellum, and specificity of cellular transduction was determined by cellular morphology and laminar distribution.

**Figure 3:**
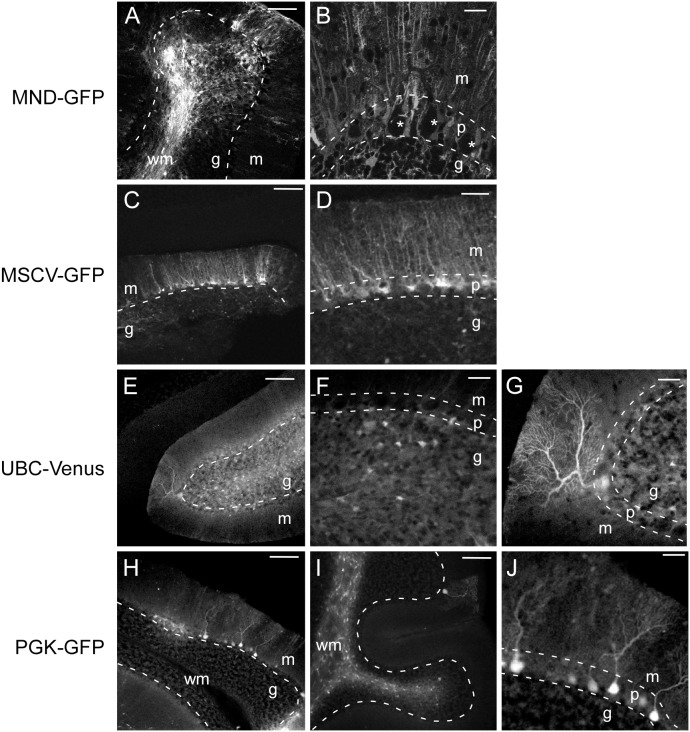
Expression of reporter genes in cerebellar cells transduced with lentiviral vectors under various promoters. Representative confocal images of sagittal cerebellar sections from mice 7–14 days following intracerebellar injection of lentiviral vectors with indicated promoters. Dotted lines demarcate the border between cerebellar cortex layers. In low magnification images (B, D, F, G, and J), the line is drawn between Purkinje and granule layers. In high magnification images (A, C, E, H, and I), two lines are drawn to separate the Purkinje layer from the molecular layer and granule layer. A. Widespread GFP expression in a cerebellar lobe injected with MND-GFP. B. Single confocal section of MND-GFP transduced cerebellum at higher magnification showing absence of GFP expression in Purkinje neuron somata (asterisks). C. Low magnification of cerebellum injected with MSCV-GFP. D. High magnification of cerebellum injected with MSCV-GFP demonstrating GFP expression in small cell bodies in the Purkinje layer with radial processes extending to the pial surface, characteristic of Bergmann glia. E. Venus expression in a cerebellar lobe injected with UBC-Venus. F, G. High magnification of UBC-Venus infected cerebellum shows venus expression in multiple small cells in the granule layer (F) and a single Purkinje neuron (G). H, I. GFP expression in cerebellar lobes of two animals injected with PGK-GFP. Several GFP-expressing Purkinje neurons are visible in H, whereas most GFP-expressing cells in I are in the white matter, with a single GFP-positive Purkinje neuron. J. High magnification view of GFP-positive Purkinje neurons from H. Abbreviations: m = molecular layer; p = Purkinje layer; g = granule layer; wm = white matter. Scale bars, 25 µm (B, D, F, G, J), 100 µm (A, C, E, H, I).

Consistent with previous findings using MND-tdTomato (Figure 2), intracerebellar injection of MND-GFP resulted in high levels of GFP expression in cells throughout all layers of the cerebellar cortex, with particularly bright expression in cerebellar white matter (Figure 3A, Table 1). Closer inspection of the Purkinje layer revealed teardrop shaped GFP-negative spaces (Figure 3B, asterisks), indicating an absence of GFP expression in Purkinje neurons. Radial processes from MND-GFP-transduced Bergmann glia were evident in the molecular layer (Figure 3B, Table 1). The MSCV-GFP vector produced GFP expression almost exclusively in Bergmann glia (Figure 2C and D, Table 1). Venus expression driven by the UBC promoter was observed in many small cells in the granule layer (Figure 3E and F, Table 1) and in an occasional Purkinje neuron (Figure 3E and G). In one animal injected with the PGK-GFP lentiviral vector several Purkinje neurons were transduced (Figure 3H and J). However, the cellular transduction pattern of PGK-GFP in a second animal resembled the transduction pattern of the MND constructs, with the vast majority of transduced cells located in the white matter (Figure 3I, Table 1).

### Intracerebellar injection technique does not affect cellular transduction pattern by MND-tdTomato lentivirus

**Figure 4:**
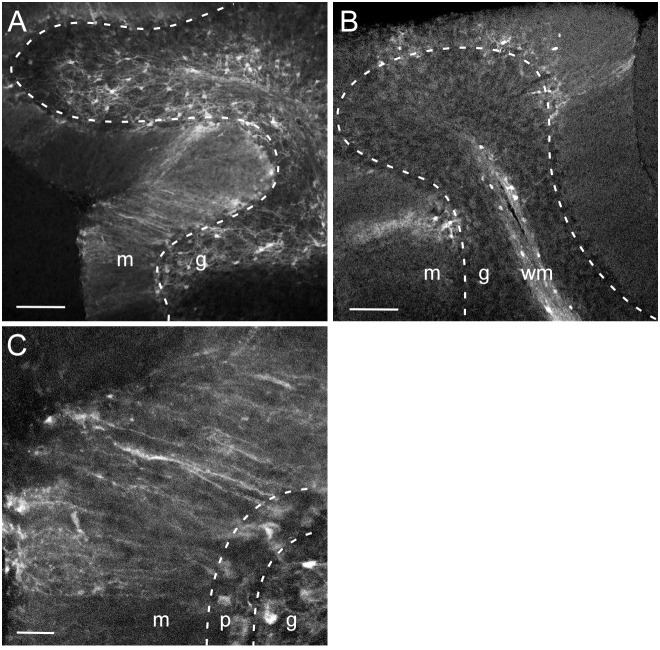
MND-tdTomato expression following injection with pulled glass pipette. Confocal images of sagittal cerebellar slices from wild type mice injected with MND-tdTomato lentivirus. Injections were performed using pulled glass pipettes and a picospritzer (see methods) to determine if injection technique affected cellular transduction pattern. Dotted lines in A and B represent the border between the Purkinje layer and granule layer. In C, dotted lines are drawn to separate the Purkinje layer from the molecular layer and granule layer. A. Widespread tdTomato expression in cells in the granule layer and processes in the molecular layer. B. A few small tdTomato expressing cells are visible in the Purkinje layer, but the majority of the tdTomato expressing cell bodies are located in the white matter (wm). C. High magnification view of tdTomato expressing cells with somata in the Purkinje layer show that their processes are relatively straight and unbranched, characteristic of Bergmann glia. m, molecular layer; p, Purkinje layer; g, granule layer. Scale bars: 100 µm (A, B); 25 µm (C).

The optimal dorsal-ventral injection depth for targeting Purkinje neurons in vivo is quite shallow, due to the relatively superficial location of Purkinje neuron somata. Intracerebellar injections up to this point were performed by injecting virus through a small burr hole in the cranium using a Hamilton syringe and nanoinjector pump. To test whether injection technique affected lentiviral cellular transduction patterns, a modified injection technique was used in which a craniotomy was performed to expose a small region of the dura, and MND-tdTomato lentiviral particles were injected using a picospritzer fitted with small diameter pulled glass pipettes (outer diameter of 18–25 µm, see methods). MND-tdTomato injected using this technique transduced many cells throughout the cerebellar cortex (Figure 4A). Similar to previous MND lentivirus injections using the Hamilton syringe (Figures 2 and 3), transduced cells were either white matter glial cells (Figure 4B) or Bergmann glia (Figure 4C). No transduced Purkinje neurons were observed, suggesting that injection technique does not affect viral transduction pattern.

### AAV serotype 1 effectively transduces Purkinje neurons and viral delivered FGF14 is properly localized

**Figure 5:**
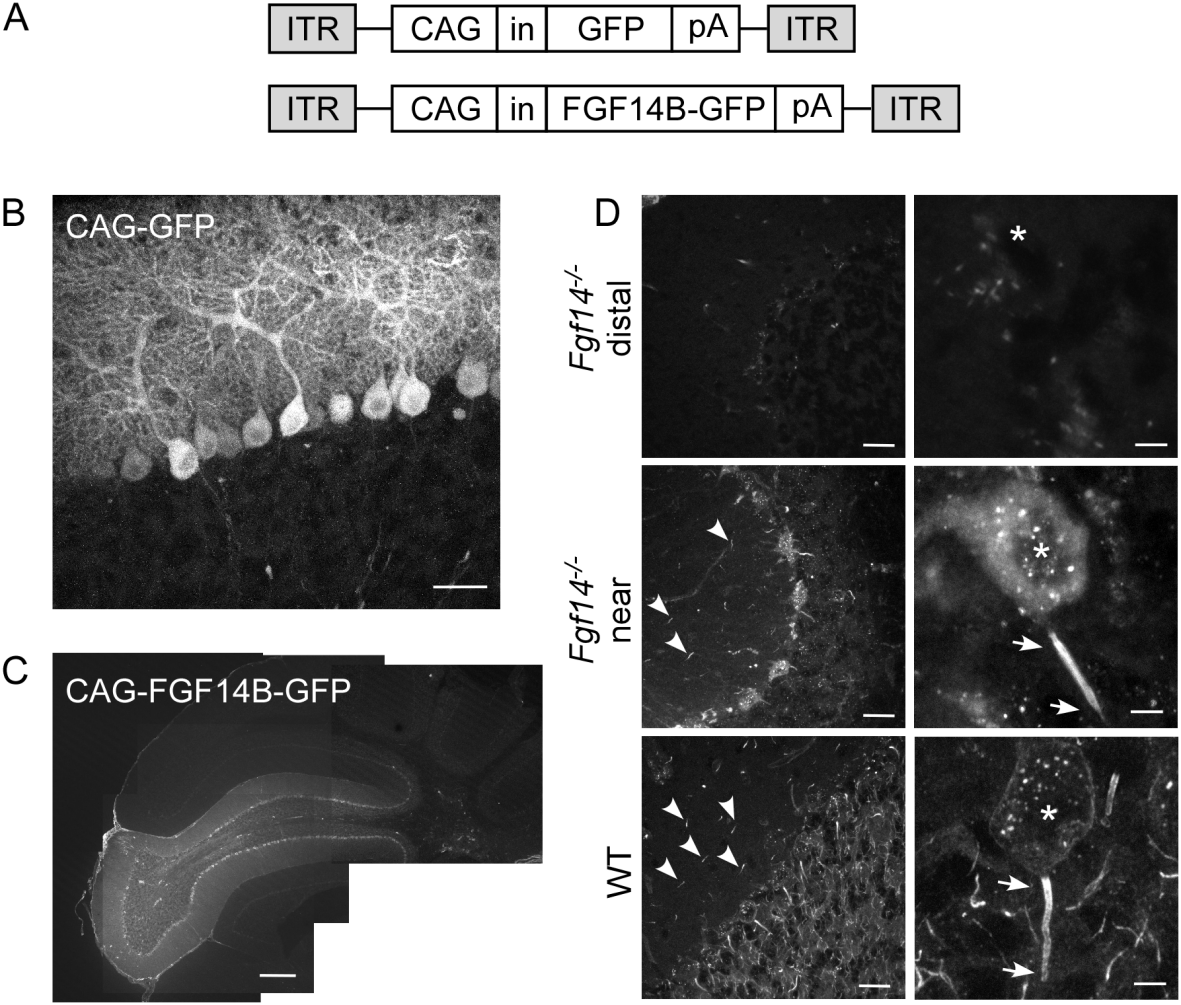
AAV1 transduction of Purkinje neurons and localization of viral delivered FGF14B-GFP. A. Schematic representation of AAV transfer plasmid constructs for AAV-CAG-GFP and AAV-CAG-FGF14B-GFP. CAG, chicken β-actin promoter with CMV enhancer; in, SV40 intron; pA, polyadylation site; ITR, inverted terminal repeat. B. Confocal image from a sagittal cerebellar section injected with AAV1-CAG-GFP showing predominant GFP expression in Purkinje neuron somata and dendrites. Some GFP expressing cell bodies are visible in the granule layer. C, D. Immunostaining for FGF14 in an *Fgf14^−/−^* mouse with intracerebellar injection AAV1-CAG-FGF14B-GFP. C. Low magnification montage of FGF14-specific immunostaining in an *Fgf14^−/−^* mouse injected with AAV-CAG-FGF14B-GFP. FGF14 expression is evident in an entire lobe. D. Immunostaining for FGF14 in an *Fgf14^−/−^* mouse injected with AAV-CAG-FGF14B-GFP shows no FGF14 expression in areas distal to the injection (top) whereas areas near the injection show rescue of FGF14 expression in Purkinje neuron soma and AIS (middle). For comparison, normal FGF14 expression in a wild type cerebellum is shown at the bottom. Asterisks mark the location of the Purkinje neuron soma and arrows mark the approximate start and end of the AIS. Arrowheads mark the AIS of FGF14-expressing stellate and basket cells in the molecular layer. Scale bars: 200 µm (C); 25 µm (B, left panels in D); 5 µm (right panels in D).

Because of very low Purkinje neuron transduction efficiency using a variety of lentiviral vectors, an alternative viral vector was sought. Previous reports suggested that adeno-associated viruses (AAV) are capable of transducing Purkinje neurons *in vivo* [33]–[35]. To assess Purkinje neuron transduction efficiency with AAV vectors, an AAV, serotype 1 (AAV1) with a CAG (chicken β-actin with CMV enhancer) promoter driving expression of EGFP (CAG-GFP, Figure 5A) was injected directly into the cerebellum of wild type mice. Three to four weeks following injection, sagittal cerebellar sections were examined, and abundant Purkinje neuron transduction was clearly evident with the CAG-GFP virus (Figure 5B, Table 1).

To determine whether viral delivered transgenes were properly targeted to subcellular domains, an AAV1 vector expressing a Fibroblast Growth Factor 14 (FGF14) with carboxyl terminal GFP fusion protein was generated (CAG-FGF14B-GFP, Figure 5A). FGF14 is enriched at the axon initial segment (AIS) of wild-type neurons [11], [26], [36], and addition of a GFP reporter gene to the carboxyl terminus does not interfere with its function or localization *in vitro* [26]. In murine cerebellum, FGF14 immunoreactivity has been reported in the Purkinje and granule layers [10], with enrichment at the AIS of Purkinje neurons [11]. To determine the expression pattern of viral delivered FGF14, FGF14B-GFP-expressing virus was stereotactically injected into the cerebellum of *Fgf14^−/−^* mice [17], and sagittal cerebellar sections were immunostained with a monoclonal anti-FGF14 antibody (see methods). Consistent with previous reports in *Fgf14^−/−^* cerebellum [10], no FGF14 immunostaining was visible in *Fgf14^−/−^* cerebellum distal to the injection site (Figure 5C and Figure 5D, top). In contrast, FGF14 immunoreactivity in *Fgf14^−/−^* mice injected with FGF14B-GFP-expressing virus was evident in an entire cerebellar lobe proximal to the injection site when examined at low magnification (Figure 5C). Closer inspection of the Purkinje and molecular layers proximal to the injection site revealed FGF14 expression in the both the AIS and somata of Purkinje neurons and the AIS of neurons in the molecular layer (Figure 5D, middle, Table 1). For comparison, anti-FGF14 immunolabeling of a wild type, non-transduced cerebellar section is included (Figure 5D, bottom), in which FGF14 expression is evident in the granule layer, Purkinje neuron AIS, and AIS of neurons in the molecular layer (Figure 5D, bottom left). In addition, wild type FGF14 was also localized to the Purkinje neuron soma membrane (Figure 5D, bottom right). No endogenous fluorescence of viral-delivered FGF14B-GFP was detectable (data not shown).

### Lack of reporter gene fluorescence in Purkinje neurons transduced with IRES and P2A containing viruses

**Figure 6:**
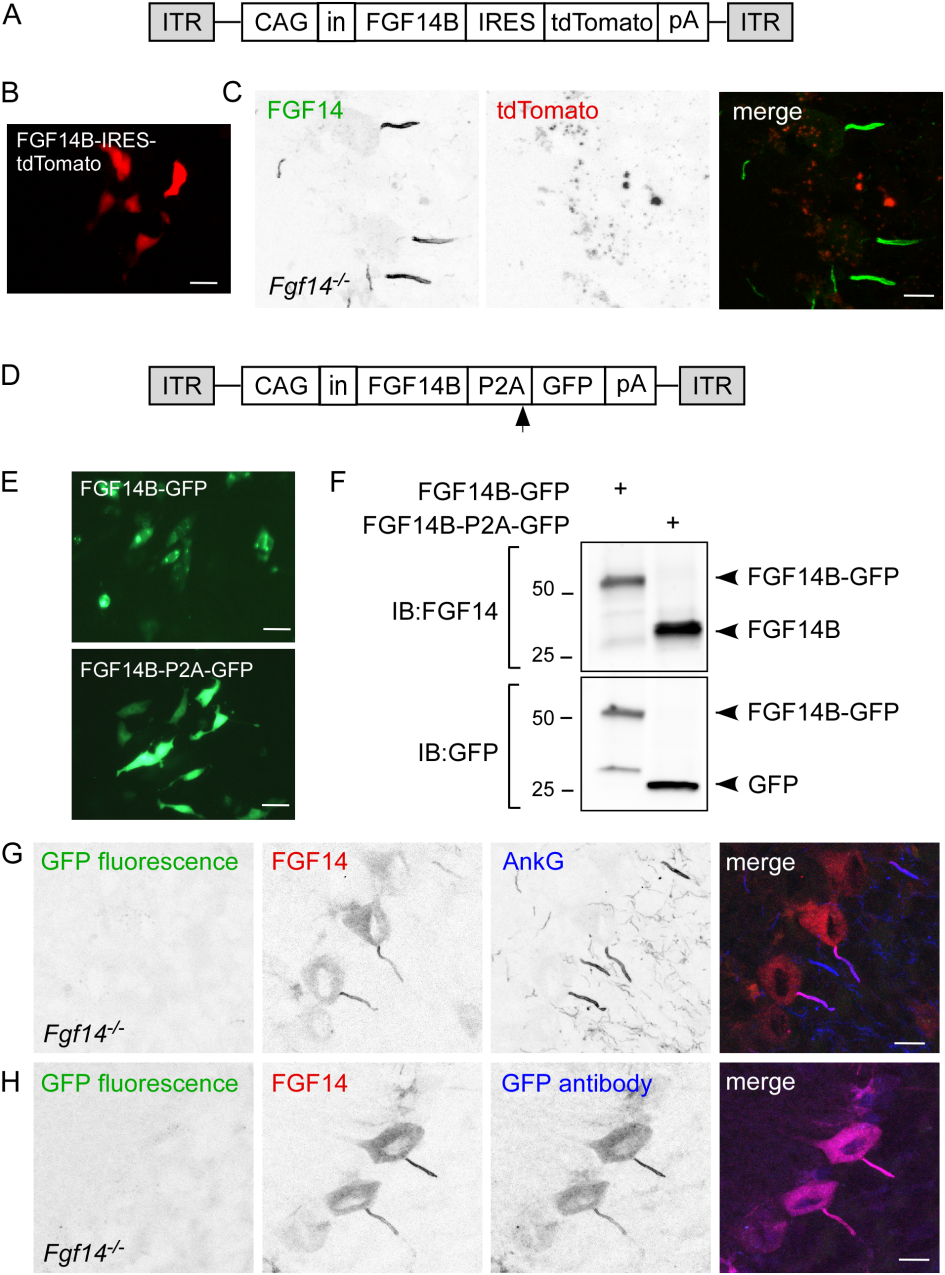
AAV1 delivery of FGF14B-IRES-tdTomato and FGF14B-P2A-GFP results in efficient FGF14 expression but failure of reporter gene fluorescence. A. Schematic representation of CAG-FGF14B-IRES-tdTomato AAV transfer construct. CAG, chicken β-actin promoter with CMV enhancer; in, SV40 intron; IRES, internal ribosome entry site; pA, polyadenylation site; ITR, inverted terminal repeat. B. CAG-FGF14B-IRES-tdTomato transfected into CHL1610 cells produces a diffuse cytoplasmic tdTomato expression pattern. C. Confocal image from an *Fgf14^−/−^* mouse injected with CAG-FGF14B-IRES-tdTomato and immunostained for FGF14. Viral delivered FGF14 is properly localized at the Purkinje neuron AIS but tdTomato expression is not visible. D. Schematic representation of CAG-FGF14B-P2A-GFP AAV transfer construct. The arrow represents approximate location where ribosomal skipping should occur to generate two independent polypeptides. E. GFP expression in CHL1610 cells transfected with CAG-FGF14B-GFP (top) or CAG-FGF14B-P2A-GFP (bottom). FGF14B-GFP fusion protein is expressed in punctate foci surrounding the nucleus whereas FGF14B-P2A-GFP is expressed as a diffuse cytoplasmic protein, suggesting cleavage of GFP from FGF14B. F. Western blot analysis of P2A cleavage efficiency in CHL cells. CHL1610 cells were transfected with either CAG-FGF14B-GFP or CAG-FGF14B-P2A-GFP and processed for western blot 24 h after transfection. Immunoblotting for both FGF14 and GFP revealed a ∼50kDa band in CAG-FGF14B-GFP transfected cells, which is consistent with the expected size of the fusion protein. Immunoblotting for FGF14 and GFP in CAG-FGF14B-P2A-GFP transfected cells revealed ∼25kDa bands for FGF14 and GFP and no detectable ∼50kDa band, indicating efficient cleavage of the P2A peptide. G. Confocal image of *Fgf14^−/−^* cerebellum injected with CAG-FGF14B-P2A-GFP and immunostained for FGF14 (red) and AnkyrinG (AnkG, blue). No GFP fluorescence is visible but viral delivered FGF14 is properly expressed at the AIS of Purkinje neurons where it colocalizes with AnkyrinG. H. Confocal image of *Fgf14^−/−^* cerebellum injected with CAG-FGF14B-P2A-GFP and immunostained for FGF14 (red) and GFP (blue). Immunostaining reveals that GFP is expressed and colocalizes with FGF14 in the Purkinje neuron AIS, suggesting that P2A cleavage did not occur *in vivo*. Scale bars: 20 µm (B, E); 10 µm (C, G, H).

Many studies would benefit from co-expression of multiple heterologous proteins in neurons. For example, electrophysiological studies of viral-transduced neurons require expression of a fluorescent reporter gene for identification of transduced cells. Since GFP fluorescence in FGF14B-GFP expressing Purkinje neurons was not visible without immunostaining, other strategies to co-express a fluorescent reporter gene in *Fgf14^−/−^* Purkinje neurons transduced with an FGF14-expressing virus were tested. Figure 6A, illustrates an AAV transfer vector in which FGF14 expression is driven by the CAG promoter and tdTomato expression is controlled by an internal ribosome entry site (IRES) downstream from FGF14B. To verify expression of tdTomato in the IRES context, CHL cells were transfected with CAG-FGF14B-IRES-tdTomato plasmid DNA, and tdTomato fluorescence was examined 24 h after transfection. As shown in Figure 6B, tdTomato was expressed in a cytoplasmic distribution in these cells. To investigate the distribution of FGF14 and tdTomato in transduced Purkinje neurons, FGF14B-IRES-tdTomato-expressing AAV1 virus was stereotactically injected into *Fgf14^−/−^* cerebellum, and sagittal cerebellar sections were immunostained for FGF14. Consistent with findings from FGF14B-GFP transduced Purkinje neurons (Figure 5), FGF14 immunolabeling was present at the AIS of FGF14B-IRES-tdTomato-transduced Purkinje neurons (Figure 6C, Table 1). Surprisingly, however, tdTomato fluorescence was not detectable (Figure 6C). In regions near the injection site, 97.9% of Purkinje neurons were transduced with the FGF14B-IRES-tdTomato virus, as demonstrated by the number of anti-FGF14 positive Purkinje neuron AIS relative to the number of anti-AnkyrinG positive Purkinje neuron AIS (Table 2).

**Table 2:** AIS expression of virally transduced FGF14 protein in *Fgf14^−/−^* Purkinje neurons.

Virus	AnkyrinG AIS^c^	FGF14 AIS^c^	% Transduced
AAV1-FGF14B-IRES-tdTomato^a^	14	13.7	97.9
AAV1-CAG-FGF14A-PGK-GFP^b^	8.1	7.9	97.5

a Average of three 60x fields near injection site.

b Average of nine 60x fields near injection site.

c Immunostaining (number of AIS stained/60x field).

2A sequences have been shown to mediate cleavage between two protein coding sequences via a ribosomal skip mechanism [9], and have been used in viral vectors to coordinate expression of two genes in neurons [4], [37], [38]. An AAV-CAG vector in which FGF14B was separated from GFP by a P2A (porcine-teschovirus-1) peptide coding sequence [27] was generated (Figure 6D). To verify fluorescence of GFP and P2A-mediated cleavage of FGF14 from GFP, the FGF14B-P2A-GFP or FGF14B-GFP (fusion) constructs (Figure 6D and 5A) were transfected into CHL1610 cells, and 24 h following transfection, cells were examined for GFP fluorescence (Figure 6E). Similar to previous reports in NIH3T3 cells [39], GFP expression in cells transfected with FGF14B-GFP plasmid was excluded from the nucleus and present in discrete punctae adjacent to the nucleus (Figure 6E, top). In contrast, in cells transfected with FGF14B-P2A-GFP, GFP expression was localized throughout the cytoplasm (Figure 6E, bottom). A similar GFP expression pattern was seen in cells transduced with virus expressing FGF14-P2A-GFP (data not shown). Following analysis of GFP expression, cells were immediately processed for Western blot analysis, and membranes were probed with anti-GFP or anti-FGF14 specific antibodies. Consistent with previous Western blot studies of FGF14B-GFP [36], immunoblotting of protein extracts from FGF14B-GFP transfected cells revealed a ∼50 kDa band when probed with the anti-GFP antibody (Figure 6F, bottom), the expected size for an FGF14B-GFP fusion protein. A ∼50 kDa band was also present in FGF14B-GFP extracts probed with the anti-FGF14 antibody (Figure 6F, top), indicating that the FGF14B-GFP fusion protein is detectable with both anti-GFP and anti-FGF14 antibodies. In stark contrast, a ∼25 kDa band was present in FGF14B-P2A-GFP extracts probed with either the anti-GFP antibody (Figure 6F, bottom) or the anti-FGF14 antibody (Figure 6F, top). No ∼50 kDa band representing FGF14B-GFP fusion protein product was evident in the FGF14B-P2A-GFP cell extracts when probed with either antibody (Figure 6F), indicating that the P2A-mediated cleavage was efficient in CHL cells.

To examine localization and cleavage of FGF14-P2A-GFP in Purkinje neurons, FGF14B-P2A-GFP-expressing virus was stereotactically injected into *Fgf14^−/−^* mouse cerebellum, and sagittal cerebellar sections were immunostained with anti-FGF14 and anti-AnkyrinG-specific antibodies. Similar to previous reports in wild type neurons [40], AnkyrinG localized to the AIS in *Fgf14^−/−^* Purkinje neurons (Figure 6G). Transduced Purkinje neurons were identified by FGF14 immunolabeling, which was present in the Purkinje neuron soma and AIS (Figure 6G, Table 1). Unexpectedly, no GFP fluorescence was visible (Figure 6G), despite the fact that an AAV-GFP virus containing the same GFP coding sequence produced robust GFP expression in Purkinje neurons (Figure 5B). To determine if GFP protein was synthesized in these cells, sagittal cerebellar sections from brains injected with FGF14B-P2A-GFP were immunostained with anti-FGF14 and anti-GFP antibodies (Figure 6H). Transduced Purkinje neurons were identified by anti-FGF14 immunolabeling (Figure 6H). Surprisingly, while no direct GFP fluorescence was visible, the presence of GFP protein was confirmed by anti-GFP immunolabeling (Figure 6H). Moreover, the subcellular localization of FGF14 and GFP immunolabeling was identical (Figure 6H), consistent with a failure of P2A-mediated cleavage in murine Purkinje neurons *in situ* and loss of fluorescent properties of the non-cleaved FGF14-P2A-GFP fusion protein.

### Transgene expression in cerebellar cells transduced with dual-promoter AAV virus

**Figure 7:**
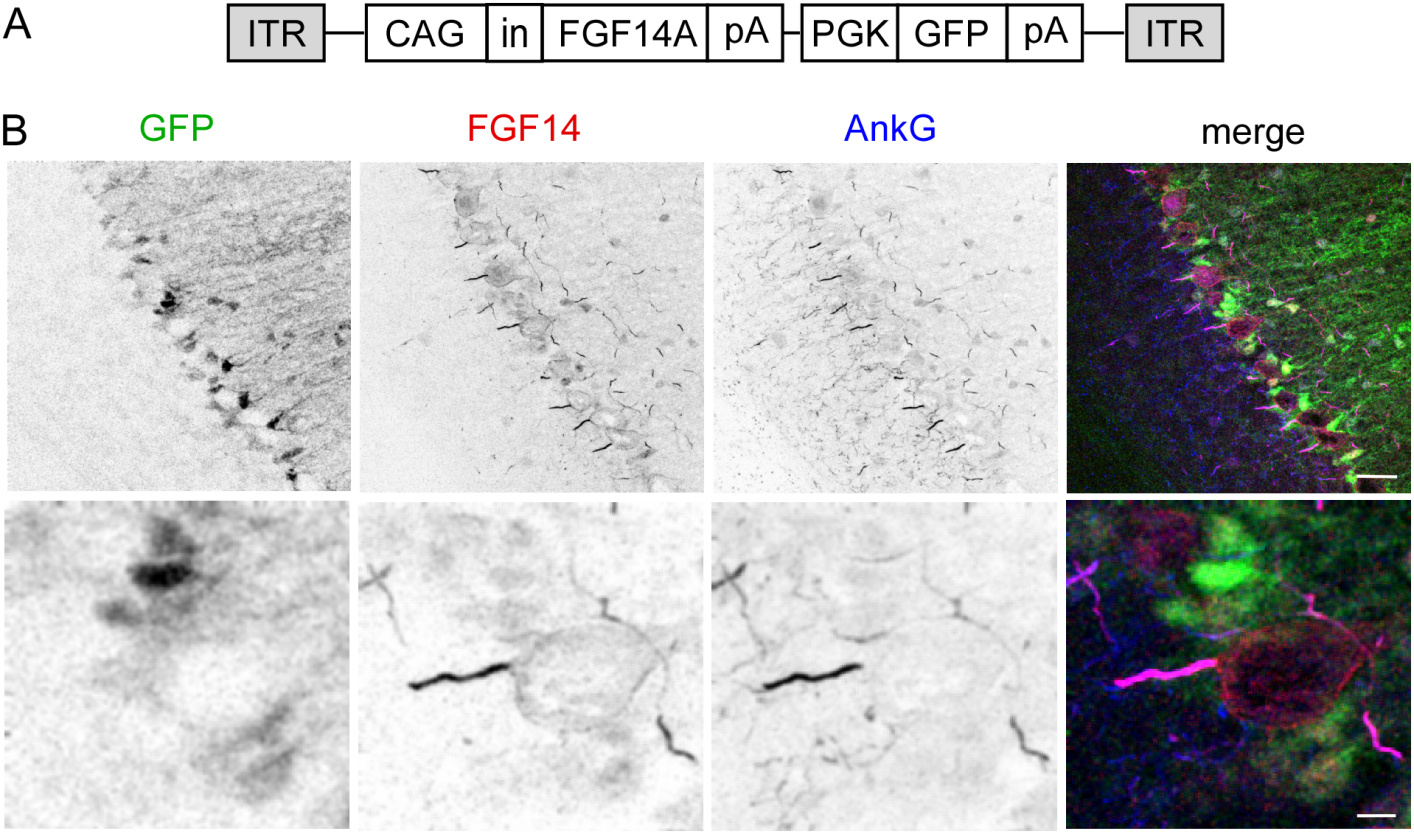
AAV1 delivery of a dual promoter construct reveals that the CAG promoter produces better Purkinje neuron expression than the PGK promoter. A. Schematic representation of the dual promoter AAV transfer construct which contains the CAG promoter followed by FGF14A and the PGK promoter followed by GFP. CAG, chicken β-actin promoter containing the CMV enhancer; in, SV40 intron; PGK, human phosphoglycerate kinase promoter; pA, polyadenylation site; ITR, inverted terminal repeat. B. Confocal images of sagittal sections from an *Fgf14^−/−^* mouse injected with the AAV-CAG-dual promoter virus and immunostained for FGF14 (red) and AnkyrinG (blue). Viral delivered FGF14 is properly localized at the Purkinje neuron AIS where it colocalizes with AnkyrinG. A lower level of FGF14 expression is visible on the soma membrane. Viral delivered GFP is expressed in small cells in the Purkinje layer that extend radial processes into the molecular layer (a pattern that is consistent with Bergmann glia) but is clearly absent from Purkinje neurons. Scale bars: 25 µm (B, top); 5 µm (B, bottom).

Viral vectors with dual promoters have also been used to drive expression of separate transgenes in neurons [5]. To determine whether FGF14 and GFP could be co-expressed in the same Purkinje neuron using a dual promoter vector, an AAV construct was generated in which FGF14A expression was controlled by the CAG promoter and GFP was controlled by the PGK promoter (Figure 7A). The PGK promoter was chosen for the second promoter because GFP was expressed in some Purkinje neurons when driven by the PGK promoter in a lentiviral construct (Figure 3H–J). *Fgf14^−/−^* mice cerebella were stereotaxically injected with CAG-FGF14A-PGK-GFP-expressing AAV1 virus, and cerebella were examined for GFP and FGF14 expression three to four weeks after injection by immunostaining sagittal sections with anti-FGF14 and anti-AnkyrinG antibodies. Similar to other FGF14 expressing viruses, transduction with CAG-FGF14A-PGK-GFP-expressing virus resulted in FGF14 immunolabeling in the Purkinje neuron AIS, where it colocalized with AnkryinG (Figure 7B, Table 1). FGF14 immunolocalization was not, however, found in the cytoplasm of the Purkinje neuron soma but was observed at the Purkinje neuron soma membrane, albeit at a lower level than at the AIS (Figure 7B). In regions near the injection site, 97.5% of Purkinje neurons were transduced with the CAG-FGF14A-PGK-GFP virus, as demonstrated by the number of anti-FGF14 positive Purkinje neuron AIS relative to the number of anti-AnkyrinG positive Purkinje neuron AIS (Table 2). Surprisingly, GFP expression was not present in Purkinje neurons but instead was robustly expressed in small cells in the Purkinje layer that extend radial processes into the molecular layer, a pattern that is consistent with Bergmann glia (Figure 7B), even though Purkinje neurons were clearly transduced, as evidenced by FGF14 immunolabeling. Conversely, no FGF14 expression was apparent in Bergmann glia, despite the fact that Bergmann glia were also clearly transduced with CAG-FGF14-PGK-GFP expressing virus (Figure 7B).

### Co-injection of two AAV1-CAG viruses allows expression of transgene and fluorescent reporter gene in the same Purkinje neuron

**Figure 8:**
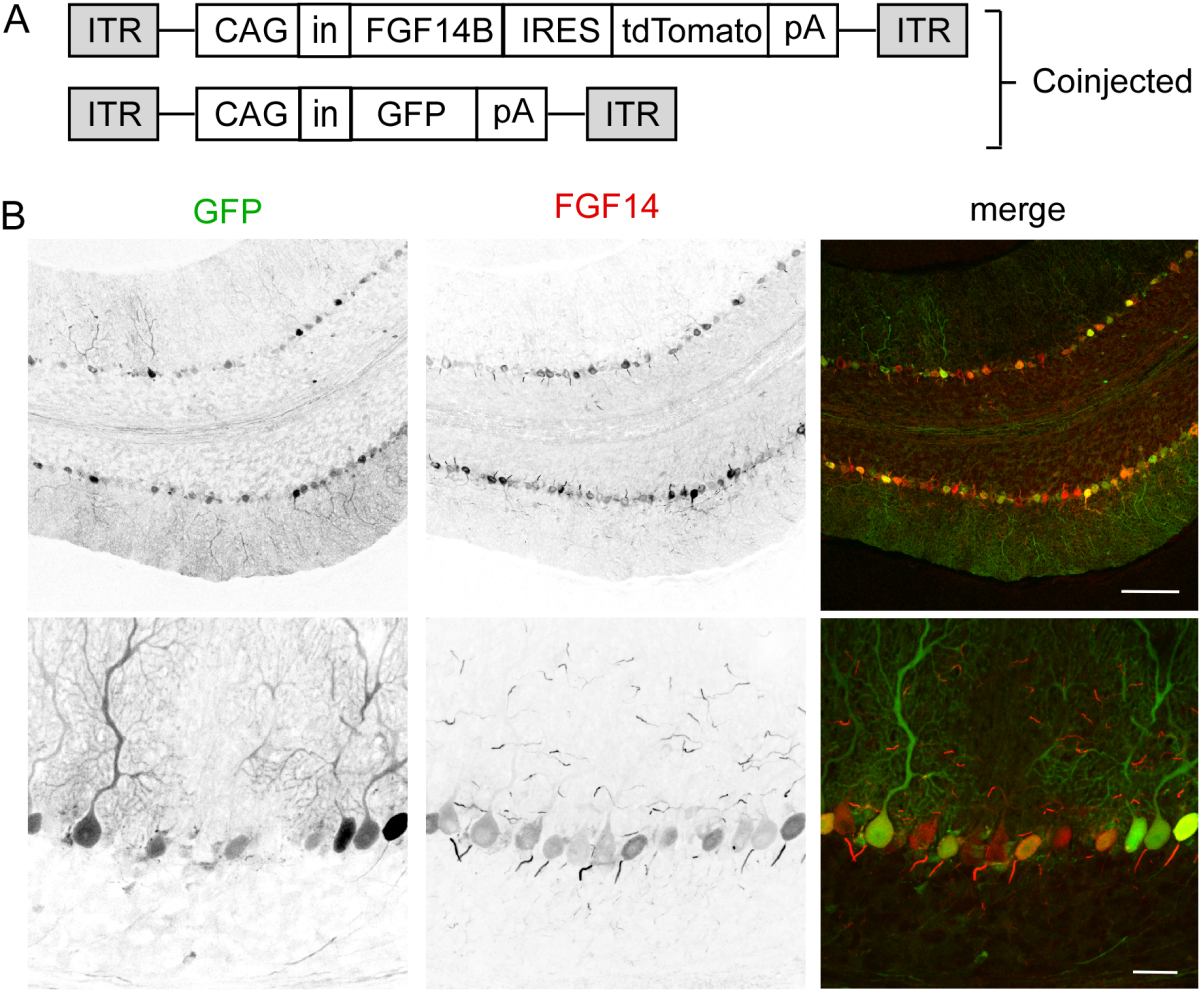
Co-injection of two AAV1-CAG viruses results in optimal Purkinje neuron expression of two different genes. A. Schematic representation of the two AAV transfer constructs used for co-injection. AAV1-CAG-GFP and AAV1-CAG-FGF14B-IRES-tdTomato viruses were mixed together at a ratio of 1∶5 prior to injecting into *Fgf14^−/−^* cerebellum. CAG, chicken β-actin promoter with CMV enhancer; in, SV40 intron; IRES, internal ribosome entry site; pA, polyadenylation site; ITR, inverted terminal repeat. B. Confocal images of sagittal sections from an *Fgf14^−/−^* cerebellum co-injected with AAV1-CAG-GFP and AAV1-CAG-FGF14B-IRES-tdTomato and immunostained for FGF14 (red). GFP expression is readily visible in Purkinje neuron somata and dendrites, and FGF14 is properly localized to the AIS. While some Purkinje neurons express FGF14 but not GFP, all GFP expressing Purkinje neurons express FGF14. Scale bars: 25 µm (B, top); 5 µm (B, bottom).

Co-injection of two viruses expressing different genetic sequences has been used to co-express multiple transgenes in the same cell [6]. To determine whether FGF14 and GFP could be co-expressed in the same Purkinje neuron when delivered by separate viruses, separate AAV1-CAG viruses expressing either FGF14B-IRES-tdTomato or GFP were used (Figure 8A). To increase the likelihood that a given cell expressing GFP would also express FGF14, viruses were mixed in a 5∶1 (FGF14:GFP) ratio and then stereotactically co-injected into *Fgf14^−/−^* cerebellum. Three to four weeks post-injection, sagittal cerebellar sections were immunostained with an FGF14-specific antibody. Consistent with previous findings in cerebella injected with a single virus (Figure 5B and 6C), GFP was expressed throughout the cytoplasm of many Purkinje neurons, and FGF14 was expressed in both the cytoplasm and AIS of Purkinje neurons (Figure 8B). Neurons in the molecular layer (stellate and basket cells) also expressed FGF14 at the AIS (Figure 8B). Multiple Purkinje neurons expressing both GFP and FGF14 were evident (Figure 8B). Whereas some Purkinje neurons expressing FGF14 alone were present, no neurons expressing GFP alone could be identified (Figure 8B, bottom), which was consistent with the relative amounts of each virus in the co-injection mix.

## Discussion

We have compared gene delivery to mature murine Purkinje neurons *in vivo* using AAV1 or lentiviral vectors with various promoter combinations and optimized for expression of two proteins in the same Purkinje neuron. Expression of fluorescent reporter proteins in lentiviral vectors appeared to be highly promoter dependent, since MND and MSCV promoters produced almost exclusively glial cell expression patterns, whereas the UBC promoter expressed well in granule neurons, and the PGK promoter appeared to express in glial cells and Purkinje neurons. Because of the low Purkinje neuron transduction efficiency using lentiviral vectors, we switched to an AAV vector and fortuitously obtained excellent Purkinje neuron transduction with the first AAV vector tested, AAV1 with the CAG promoter.

Subsequent experiments designed to co-express two proteins in the same Purkinje neuron with an IRES sequence, P2A sequence, or dual promoters were unsuccessful, but we showed that two AAV1-CAG viruses were capable of efficiently transducing and expressing different transgenes in the same Purkinje neuron when co-injected. The results of this study will be useful for future studies of Purkinje neuron physiology, which require genetic manipulation of mature cells *in vivo*.

Transduction of cerebellar cells *in vivo* with lentivirus has been reported by other groups. Intracerebellar injection of lentivirus with a ubiquitous promoter from cytomegalovirus (CMV) and VSV-G envelop protein transduced a wide range of cerebellar cells including glia and Purkinje, stellate, and golgi neurons [41]. A recombinant feline immunodeficiency virus (FIV) containing a CMV promoter transduced Purkinje neurons and stellate and basket neurons in the molecular layer but few glia cells [33]. Takayama and colleagues compared VSV-G pseudotyped, HIV-derived lentiviral vectors containing promoters from MSCV, CMV, CAG, or Rous sarcoma virus (RSV), and found that the MSCV promoter produced the most efficient transduction of Purkinje neurons [42], [43]. In stark contrast, our study showed that VSV-G pseudotyped lentivirus with MSCV promoter was expressed almost exclusively in Bergmann glia. It has been reported that transduction efficiency of lentivirus with MSCV promoter shifted from Purkinje neurons to Bergmann glia when viruses were exposed to lower pH during harvest from packaging cells, and it was speculated that lower pH activates a proteolytic mechanism which alters the VSV-G envelop protein and cellular tropism [44]. Recently, Goenawan and Hirai reported that addition of Cathepsin K inhibitor to the lentiviral culture media modulated lentiviral tropism for Purkinje neurons [45]. It is possible that alterations in our lentiviral production technique could increase Purkinje neuron transduction efficiency. However, the fact that all of our lentiviruses were harvested using the same protocol but expression patterns varied depending on promoter, argues for a promoter-dependent mechanism for preferential Purkinje neuron expression. Indeed, a multitude of studies have shown that choice of promoter is critical to obtain cell-specific expression with VSV-G pseudotyped lentivirus [42], [46]–[49].

We also explored the impact of injection technique on Purkinje neuron transduction efficiency. Lenviral injections performed using a Hamilton syringe and nanoinjector pump produced a similar transduction pattern as injections performed using small diameter pulled glass pipettes and a picospritzer, suggesting that injection technique does not significantly affect Purkinje neuron transduction efficiency. In contrast, depth of injection may alter transduction pattern. Dodge et al reported that injection of various AAV serotypes into the deep cerebellar nuclei (DCN) of adult mice yielded widespread transduction of cells throughout the cerebellum, brain stem, midbrain, and spinal cord [50]. Another study found that AAV2 injected into either the DCN or cerebellar cortex transduced substantial numbers of Purkinje neurons, but that more Purkinje neurons were transduced with injections into the cerebellar cortex [34]. We did not test DCN injections under our conditions, but because our goal was to selectively transduce Purkinje neurons (versus neurons in other brain regions), cerebellar cortical injections were likely appropriate.

The relative contribution of vector promoter versus virus type or AAV serotype to Purkinje neuron specificity is unclear and difficult to assess. In our study, intracerebellar injection of AAV1 containing the CAG promoter produced transgene expression in many Purkinje neurons but also in some stellate and basket cell interneurons in the molecular layer. In a previous study, AAV5 containing the Rous sarcoma virus (RSV) promoter was found to produce transgene expression in Purkinje, stellate, and basket neurons and in a few glial cells [33]. In comparison, lentiviral particles containing the RSV promoter transduced a high percentage of glial cells but very few Purkinje neurons [42]. Kaemmerer et al. reported that AAV2 containing CMV promoter only transduced Purkinje neurons if it was co-injected with adenovirus 5 (Ad5) as a helper virus, whereas AAV2 containing the CAG promoter was highly effective at transducing Purkinje neurons [34], suggesting that the CAG promoter is perhaps more specific for Purkinje neurons than the CMV promoter. However, lentiviruses containing the CAG promoter were not specific for Purkinje neurons [42]. In contrast, the CMV promoter contained in an AAV1 vector appeared to be highly specific for Purkinje neurons, at least when viewed at low magnification [35]. CMV promoter-containing lentiviruses, either derived from HIV [42] or FIV [33], were not specific for Purkinje neurons, and while the HIV-derived lentiviruses transduced many glial cells [42], the FIV-derived lentiviruses transduced mainly neurons [33]. Cell specific expression is likely a combined result of viral particle interaction with host cell-surface factors in addition to the activity of the promoter in a given cell type. Determining the relative contributions of viral tropism versus promoter activity to gene expression following injection of virus into the cerebellum would require a careful analysis of each factor.

In our *in vivo* experiments, the FGF14B-GFP fusion protein failed to fluoresce, in contrast to its fluorescence in heterologous cells and in cultured hippocampal neurons [26], [36]. In addition, the FGF14B-P2A-GFP virus, which we hypothesize to have also produced an FGF14B-GFP fusion protein, also failed to fluoresce *in vivo*. It is clear that the message and protein is being made at a reasonable level, since the protein is detectable by antibody staining. One possible explanation for lack of endogenous GFP fluorescence could be that localization at the AIS masks the endogenous fluorescence, possibly due to interactions with other proteins.

Our results using the dual promoter AAV1 virus, in which PGK drives expression of GFP and CAG drives expression of FGF14, indicate that AAV1 does transduce both glia and neurons, but that neuronal expression is dependent on the promoter, since PGK drives expression mainly in Bergmann glia whereas CAG drives expression mostly in Purkinje neurons. Both cell types (neurons and glia) were clearly transduced by the same dual promoter virus, so it is unclear why dissociated gene expression was observed. One possibility is that the PGK promoter is less active in neurons; however, our study using lentivirus containing the PGK promoter indicated that PGK could, in fact, drive expression in Purkinje neurons. Another possibility is that the CAG promoter suppressed PGK promoter expression in Purkinje neurons by a transcriptional interference or promoter competition mechanism [51], [52]. As an alternative to using different promoters to drive expression of each transgene, we could have used two CAG promoters in tandem. We chose not to do so to avoid the potential problem of DNA recombination, either in viral packaging cells or in transduced cells. However, it is possible that we could have achieved co-expression of FGF14 and GFP using this strategy.

IRES sequences have been used for multiple gene expression in CNS neurons; however, in many instances, only the first gene is expressed strongly and IRES-dependent translation is much weaker [53]. In our hands, IRES-dependent tdTomato fluorescence was undetectable *in vivo*, even though it was robustly expressed *in vitro*. Other groups have also reported instances in which fluorescence of the second, IRES-dependent gene was undetectable [54]. Furthermore, the efficiency of IRES-dependent translation has been shown to vary in different cell types [55]. Thus, it is possible that IRES sequences are simply less efficient in Purkinje neurons; however, other groups have reported co-expression of two genes in murine Purkinje neurons using IRES sequences[56]. Further experimentation, such as antibody staining, would be required to determine whether tdTomato protein was expressed in cells transduced with our IRES-tdTomato virus.

Another method for expressing multiple proteins from a single promoter is to use 2A sequences inserted between the coding regions for each protein. 2A sequences, which induce cleavage by a ribosomal skip mechanism [9], function in all eukaryotic systems tested to date, and have become popular due to their small size and yield of essentially equimolar quantities of each protein product [8]. In the present study, we used a 2A sequence from porcine teschovirus (P2A), which was reported to produce the highest cleavage efficiency compared to other 2A sequences in multiple mammalian cell types, including mouse liver *in vivo* [27]. P2A sequences have also been used in lentiviral vectors to express multiple proteins in rat Purkinje neurons [57]. In our hands, P2A-mediated cleavage between FGF14 and GFP was quite efficient in CHL cells; however, there was no apparent cleavage in Purkinje neurons *in vivo,* as suggested by the exact co-localization of the FGF14 and GFP immunostaining at the AIS. A possible explanation for lack of cleavage could be that the P2A nucleotide sequence we used [27], differs slightly from the P2A sequence used by Ohashi and colleagues [57], although the resultant peptide sequences are identical. However, since 2A-mediated cleavage is thought to be a co-translational process in which a peptide bond is “skipped” between the Gly and Pro in the 2A motif (D(V/I)EXNPGP), most of the peptide sequence would have already been generated by the time the skipped peptide bond was reached, and it is unlikely that any small changes in nucleotide sequence would have made a difference. Other studies have shown that certain peptide sequences upstream of 2A may prevent cleavage [58], [59]. In particular, failure of 2A-mediated cleavage resulted when some secreted proteins [59] or proteins targeted to the endoplasmic reticulum (ER) [58] were placed upstream of the 2A sequence. We placed FGF14 upstream of P2A in our vector, and while FGF14 (a non-secreted protein) is cytoplasmic in HEK293 cells [39], in neurons it appeared to be localized to the membrane of the soma and AIS. Whether FGF14 is processed in the ER is unknown, but inhibition of 2A-mediated cleavage in neurons could be due to neuron-specific membrane trafficking of FGF14. Reversing the order of transgenes relative to the 2A sequence may overcome the inhibition of 2A-mediated cleavage. Other studies have indicated that placing a flexible Gly-Ser-Gly spacer, furin proteinase cleavage site, or amino acids from protein 1D immediately upstream of the 2A sequence improves cleavage efficiency [60]–[62]. Placing tandem 2A sequences between the two transgenes may also improve cleavage efficiency. We did include the Gly-Ser-Gly spacer in our construct, but we have not tested the efficacy of other spacers.

In conclusion, we have demonstrated that murine Purkinje neurons can be transduced by AAV1 vectors containing CAG promoters, and that co-injection of two CAG containing AAV1 viruses results in co-expression of two transgenes in many Purkinje neurons. The failure of many of our attempts to co-express two transgenes in Purkinje neurons highlights the importance of examining trangenes and delivery methods in the cellular context in which they will be used.
